# Ascending aorta pseudoaneurysm simulating mediastinal lymphoma in computed tomography, a possible diagnostic error: a case report

**DOI:** 10.1186/s13256-020-02465-y

**Published:** 2020-09-25

**Authors:** Marcello Chiocchi, Luigi Spiritigliozzi, Federica Di Tosto, Leonardo Benelli, Francesca D’Errico, Matteo Presicce, Luca Pugliese, Francesca Ricci, Vincenzo De Stasio, Carlo Di Donna, Monia Pasqualetto, Dionisio Ferdinando Colella, Roberto Floris

**Affiliations:** 1 Department of Diagnostic Imaging and Interventional Radiology, Policlinico Tor Vergata, Rome, Italy; 2 Cardiothoracic Anesthesiology PTV Foundation, “Tor Vergata” Hospital University of Rome “Tor Vergata” Viale Oxford, 81-00133 Rome, Italy

**Keywords:** Aortic pseudoaneurysm, Marfan syndrome, Computed tomography, Transesophageal echocardiogram, Mediastinal lymphoma

## Abstract

**Background:**

An ascending aortic pseudoaneurysm is a severe and rare complication following cardiothoracic surgery. This case report demonstrates its possible misinterpretation and the consequent importance of multidisciplinary evaluation.

**Case presentation:**

We present a case of an 18-year-old Caucasian man with Marfan syndrome who developed an ascending aortic pseudoaneurysm about 1 year after undergoing cardiac surgery with the Bentall procedure. Computed tomographic examination of the thoracic aorta and positron emission tomography–computed tomography initially suggested a lymphomatous pathology. However, these imaging results were in contrast to the transesophageal echocardiogram and the laboratory data that showed negative results for hematological pathology. A second computed tomographic scan redirected the diagnosis toward a pseudoaneurysm.

**Conclusion:**

This case demonstrates the utility of close communication and interdisciplinary consultation between cardiovascular radiologists and the cardiac surgery team, which are mandatory in order to maximize their diagnostic skills in identifying postoperative complications.

## Introduction

An ascending aortic pseudoaneurysm (AAP) is a severe and rare complication following cardiothoracic surgery. Patients present with fever, chest pain, or mass effect, but it is also possible to find a pseudoaneurysm upon imaging evaluation in asymptomatic patients [1, 2]. Recent literature does not offer definitive guidelines for the management of pseudoaneurysm, but surgical treatment is usually recommended in symptomatic as well as asymptomatic patients [3]. In this report, we present a case of an 18-year-old man with Marfan syndrome who developed AAP about 1 year after undergoing cardiac surgery with the Bentall procedure. Computed tomography of the thoracic aorta and positron emission tomography–computed tomography initially suggested lymphomatous pathology. However, the results of the above-mentioned techniques were in contrast to the transesophageal echocardiogram (TEE) and the laboratory data, which showed negative results for hematological pathology. A second CT scan redirected the diagnosis toward a pseudoaneurysm.

## Case presentation and discussion

An 18-year-old Caucasian man with Marfan syndrome came to our emergency room for treatment of syncope. About 1 year earlier, he had undergone cardiac surgery with the Bentall procedure for the treatment of aortic root dilation.

**Fig. 1:**
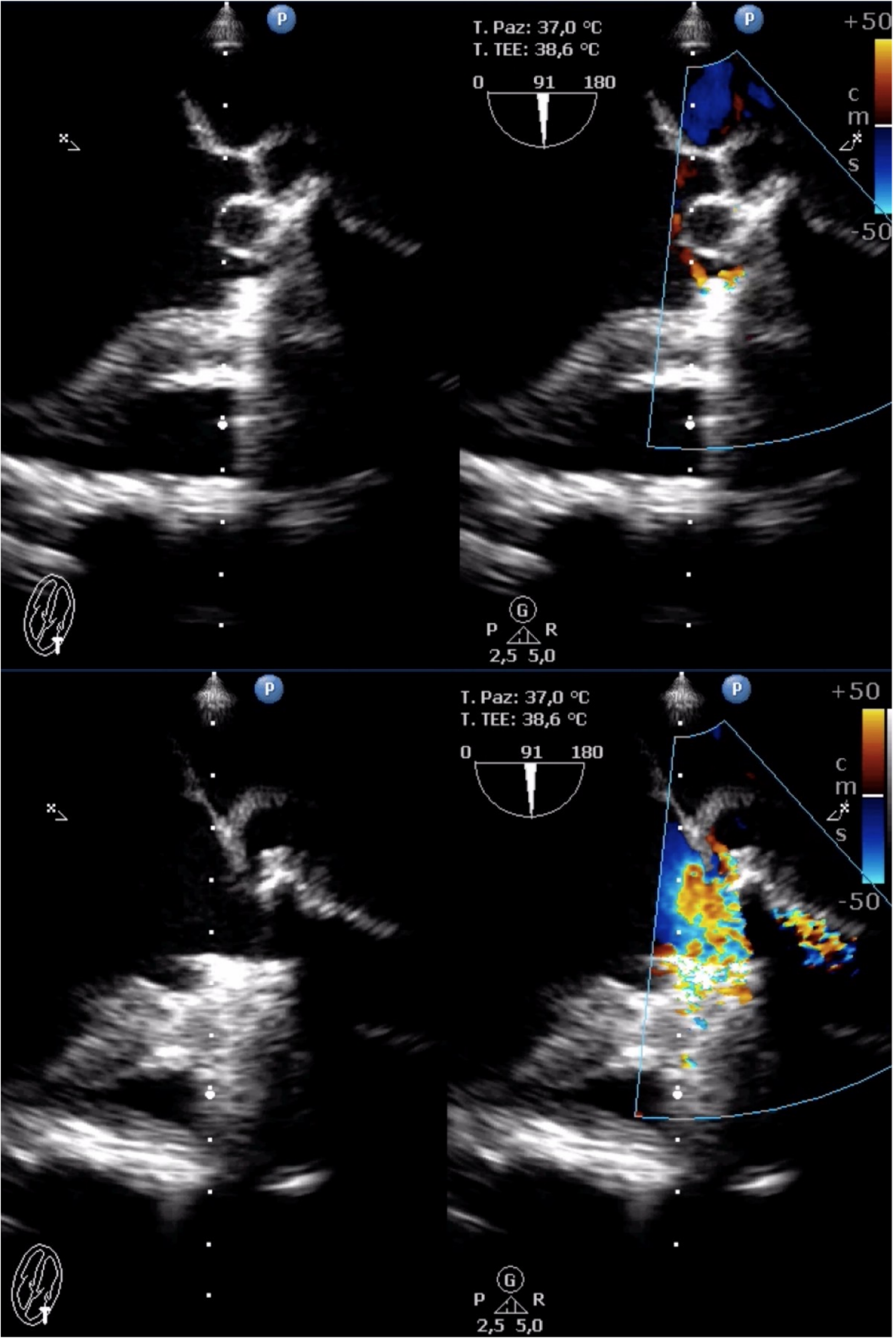
Transthoracic echocardiogram showing a hypoanechoic formation at the level of the aortic root in the periprosthetic area, which extended from the subannular plane into the periprosthetic site with apparent compressive effect on the first tract of the ascending aorta

His blood count showed a slight reduction in hemoglobin values (11.9 g/dl; baseline hemoglobin 14 g/dl) suspicious for bleeding; consequently, the administered antiplatelet therapy was suspended, and transthoracic echocardiography was performed. The examination showed a hypoanechoic formation at the level of the aortic root in the periprosthetic area, which extended from the subannular plane into the periprosthetic site with apparent compressive effect on the first tract of the ascending aorta, for which a further diagnostic investigation was considered mandatory (Fig. 1).

**Fig. 2:**
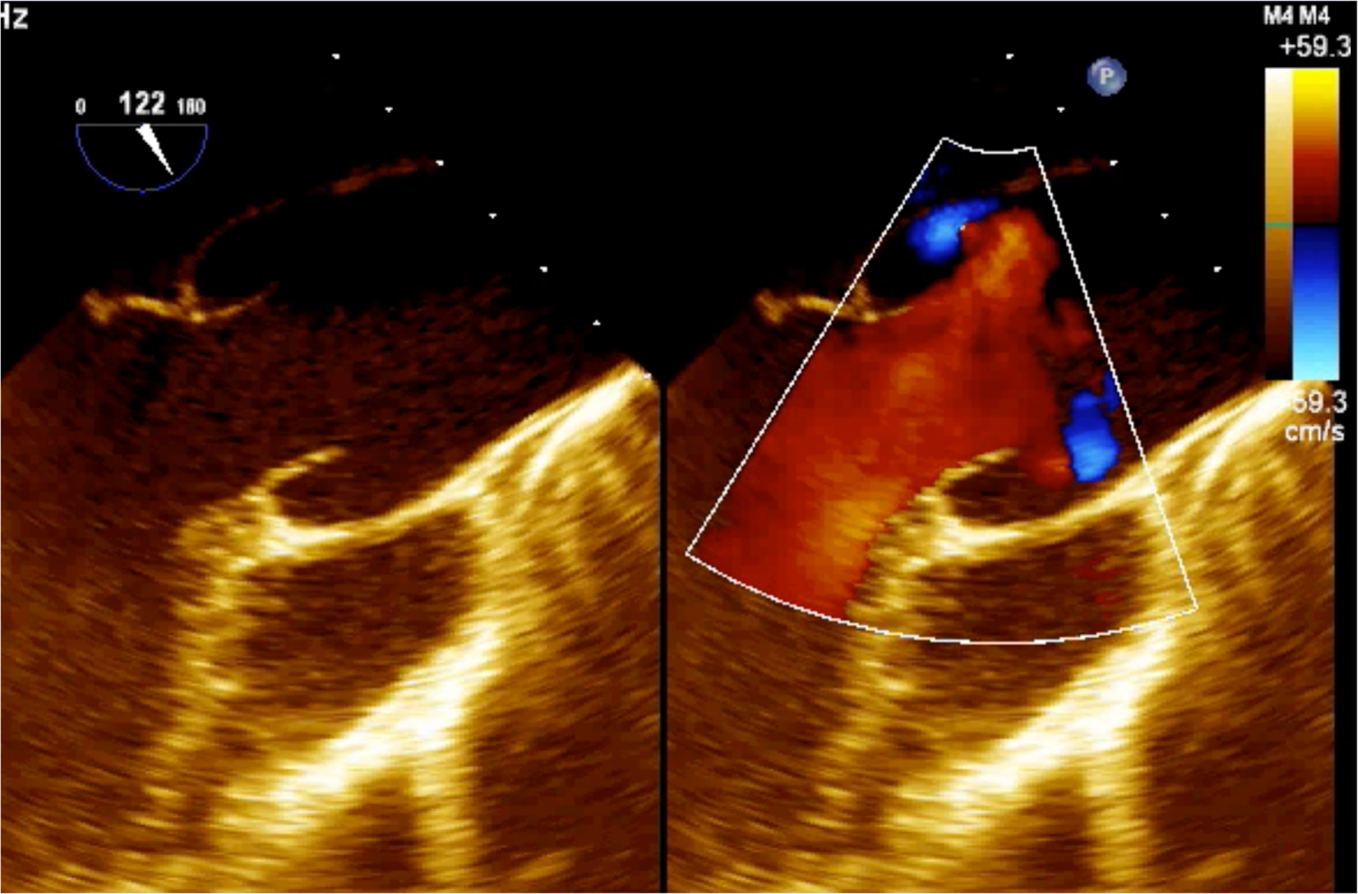
Transesophageal echocardiogram showing that the subannular anechoic formation was flattened at each systolic outflow in the absence of flow signals at the color Doppler evaluation

TEE was performed and showed that the subannular anechoic formation of uncertain diagnostic interpretation, presumably fluid, was flattened at each systolic outflow in the absence of flow signals by color Doppler evaluation (Fig. 2). Therefore, on the basis of the clinical and echocardiographic suspicion of pulsating hematoma or postoperative pseudoaneurysm, CT of the thoracic aorta was required for further characterization.

**Fig. 3:**
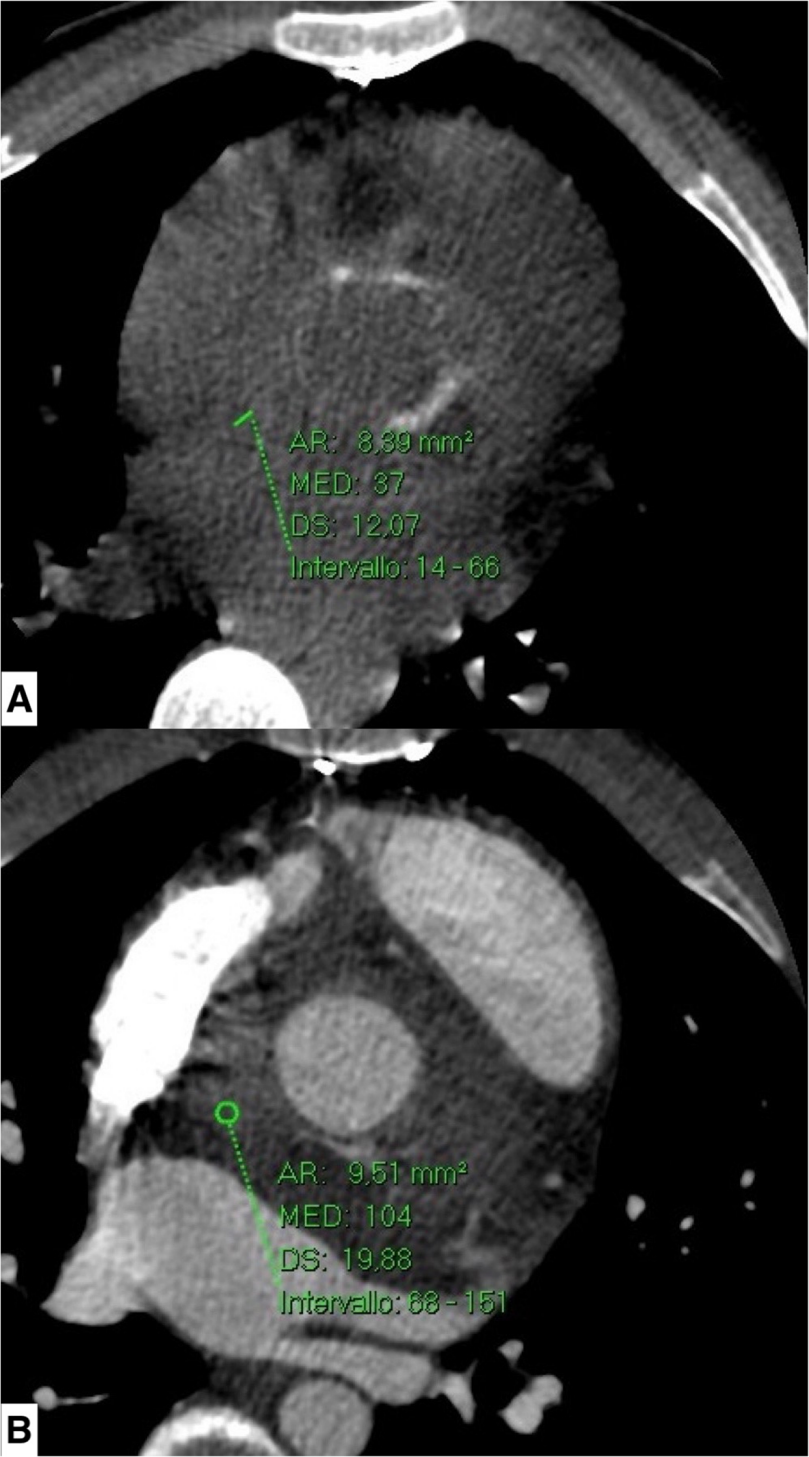
Computed tomographic scan. Hypodense area in the basal scan (**a**) in the periprosthetic site at the level of the anterior mediastinum, not characterized by significant contrast enhancement in the only arterial acquisition phase (**b**) carried out (HU (Hounsfield Unit) 37 without contrast medium; HU (Hounsfield Unit) 104 in arterial phase)

The CT examination confirmed the presence, in basal conditions, of a voluminous hypodense area in the periprosthetic site at the level of the anterior mediastinum, which, after contrast media (CM) administration, was not characterized by a moderate contrast enhancement (CE) in the only arterial acquisition phase carried out (HU (Hounsfield Unit) 37 without CM; HU (Hounsfield Unit) 104 arterial phase) (Fig. 3). Delayed images did not show either slow endoleak or significant CE of the examined area.

Because of the absence of significant CE, the radiologist required a cardiac magnetic resonance (CMR) examination under the suspicion of neoplasm in order to further investigate and characterize the nature of the lesion. However, this examination was not diagnostic because of the ferromagnetic artifacts due to the presence of the aortic prosthesis.

**Fig. 4:**
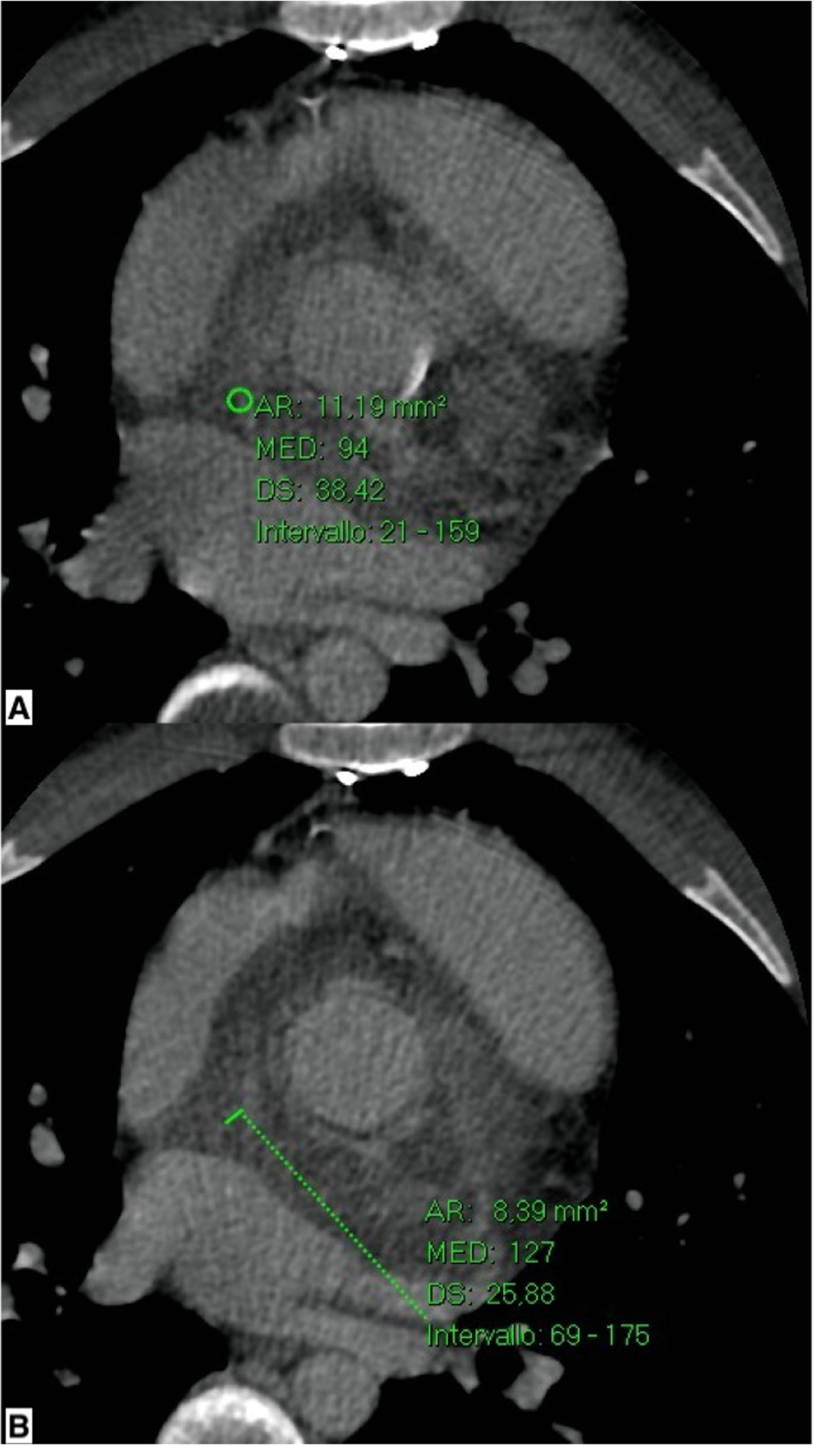
Computed tomographic scans. **a** Periaortic area shows maximum enhancement, 127 HU (Hounsfield Unit) in portal phase (70 seconds) with slow and progressive washout in late phase (**b**; 94 HU (Hounsfield Unit))

Hence, to study the nature of the suspected periprosthetic tissue, a new CT scan of the thoracic aorta was obtained with triphasic acquisition, and it showed a slow and progressive CE after CM administration with initial enhancement in portal phase (70 seconds, HU (Hounsfield Unit) 127) and full enhancement in the late phase (3.5 minutes; HU (Hounsfield Unit) 94 late phase) (Fig. 4). The presumed periprosthetic tissue had transverse dimensions of 4.5 cm and a craniocaudal extension of 6.5 cm and was characterized by a minimal overhang in the efflux tract. An enlarged lymph node of about 2 cm (short axis 1.2 cm) at the level of the aortopulmonary window was also noted.

**Fig. 5:**
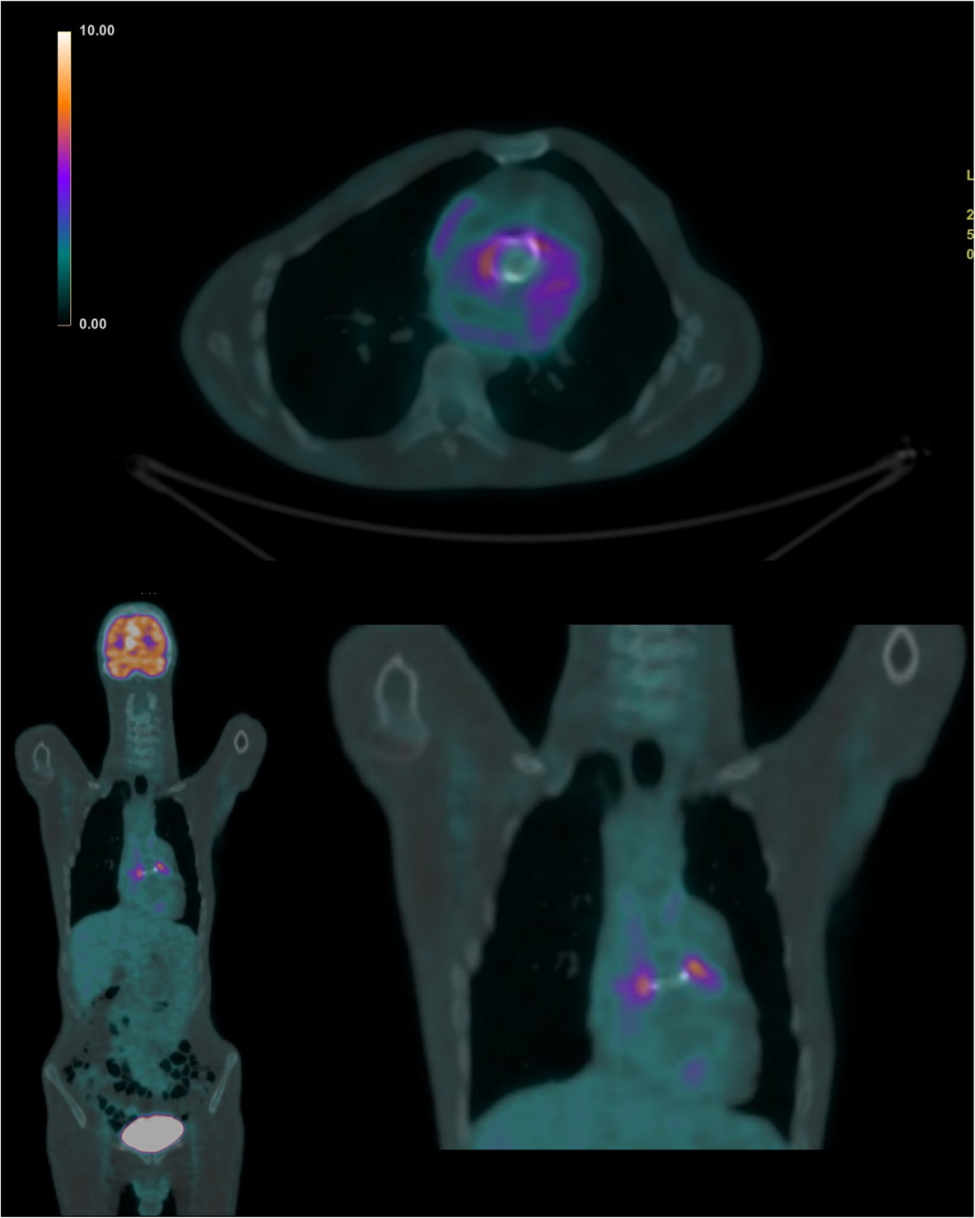
Positron emission tomography–computed tomography showing focal and diffuse uptake of the radiometabolic tracer around the tissue in the periaortic site

On the basis of post-CM behavior of the described findings, we suspected a neoplasm of probable lymphomatous nature, and consequently mediastinoscopy was suggested [4]. After a few days, PET-CT with ^18^F-fluorodeoxyglucose was performed in order to evaluate the potential uptake of the mentioned tissue. The examination showed focal and diffuse uptake of the radiometabolic tracer (standardized uptake value 10.05) around the tissue in the periaortic site, next to the aortic valve prosthesis, and in the precarinal lymph nodal station, reinforcing the hypothesis of a lymphomatous mass, and there was no further significant lymph node station uptake (Fig. 5).

The usefulness of histological characterization was then confirmed. However, surgeons were hesitant to perform a biopsy, which would have been complicated, considering the clinical history of the patient [5].

**Fig. 6:**
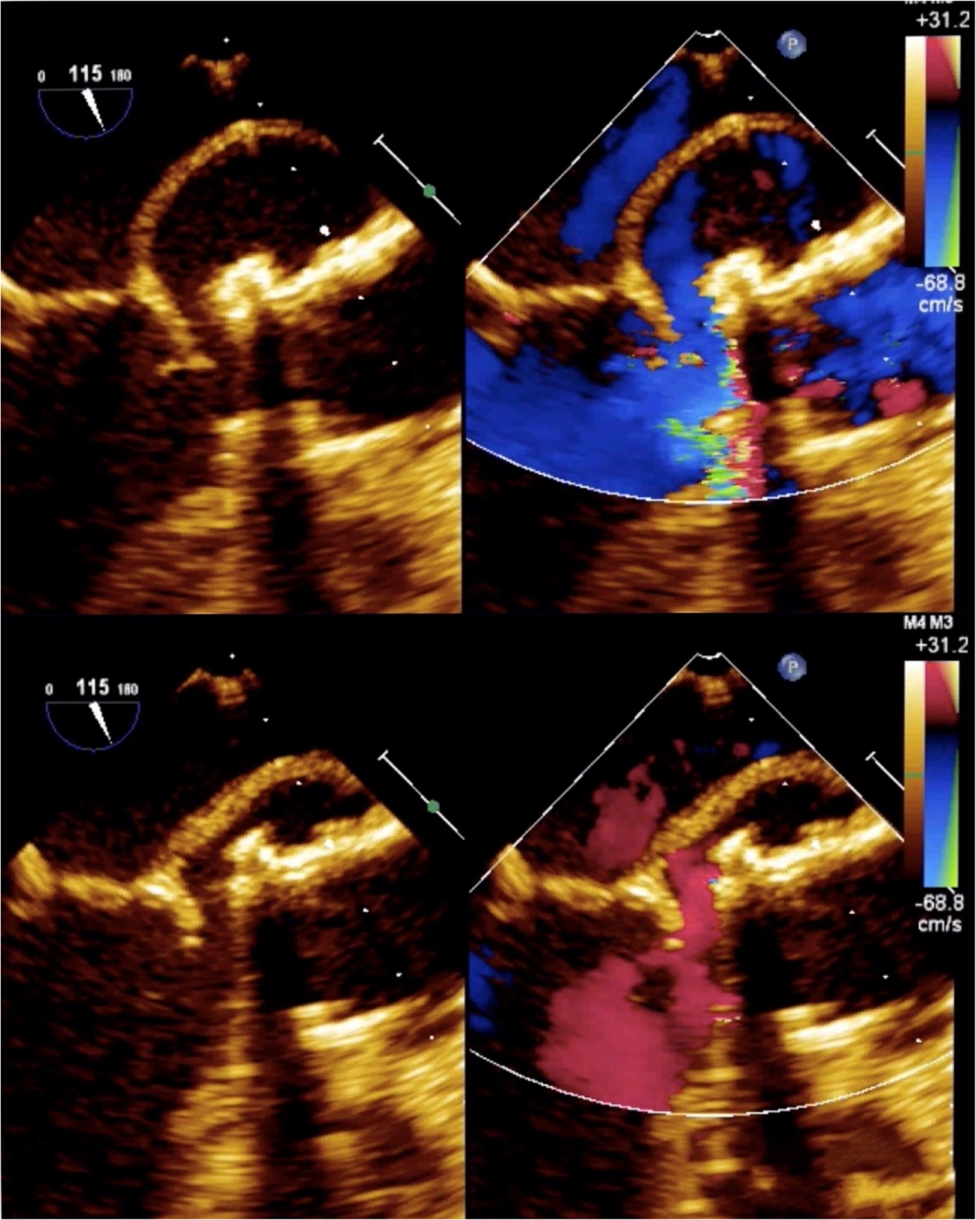
Transesophageal echocardiogram confirming the presence of the subannular anechoic formation with the appearance of internal flow signal at the color Doppler evaluation, suggesting the communication with the remaining periprosthetic sac, compatible with a refurbished pseudoaneurysm

Considering this diagnostic information provided by triphasic CT and PET-CT, daily readministration of antiplatelet therapy was allowed. Besides, the hematological consultation did not support the hypothesis of hematological disease, so the patient was redirected to a cardiac surgery consult. One week after the first TEE, a new focused TEE confirmed the presence of the subannular anechoic formation with the appearance of internal flow signal at the color Doppler evaluation, suggesting the communication with the remaining periprosthetic sac, compatible with a refurbished pseudoaneurysm (Fig. 6).

**Fig. 7:**
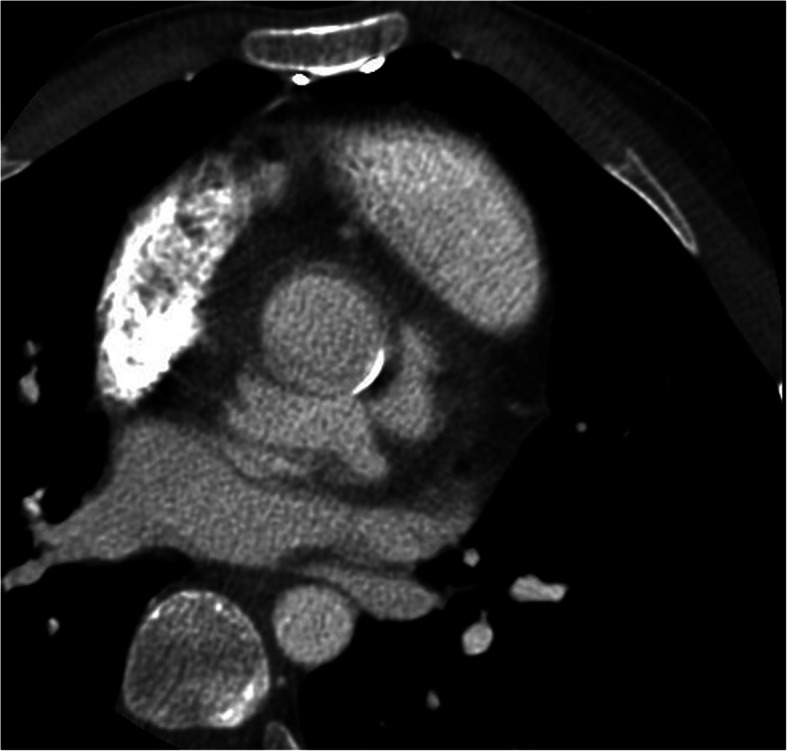
Complete and rapid filling of the periaortic hypodense area corresponding to the perianastomotic pseudoaneurysm

So, a new CT was then performed, which showed an almost complete and rapid filling of the periaortic hypodense area, confirming the diagnosis of the perianastomotic pseudoaneurysm (transverse diameter 5 cm, craniocaudal extension 4 cm) (Fig. 7). Cardiac surgeons performed a reintervention on the patient that confirmed the CT diagnosis of pseudoaneurysm [1–3, 6].

AAP is a rare complication that occurs in less than 0.5% of all cardiothoracic surgery cases at previous anastomotic sites, cannulation sites, and/or on the aortotomic suture line. It has been estimated that in about 3.3–10% of pseudoaneurysm cases, there is a hereditary connective tissue disorder such as Ehlers-Danlos syndrome and Marfan syndrome. The incidence of AAP is unclear, and about half of the AAP cases develop within 2 years from surgery [3].

The CT study, mainly using multiplanar reconstruction with 3D volume rendering, provides essential information for the subsequent therapeutic surgical planning [7, 8]. The CMR examination allows evaluation for the presence of blood or thrombotic material in the pseudoaneurysm, as well as its size and relations, and to establish its blood nature, comparing the CE intensity with that of the adjacent arteries [9, 10]. TEE is useful, but it can provide false-negative results [11].

The interesting data resulting from our case concern the noncorrespondence between the echocardiographic results and CT information. If the TEE was suspicious for a postoperative pseudoaneurysm, the CT suggested instead the presence of a tissue formation due to the following characteristics:

- The blood sac was not typically hyperdense in basal scans.
- After CM administration, no filling was observed in the arterial phase.
- In the triphasic CT study, the content of the sac had a slow and progressive parenchymatous enhancement.

Our patient’s case was discussed in a multidisciplinary team, and we believe that there are at least two different elements that may explain similar behavior of the pseudoaneurysm during CT. On the one hand, the suspension of the antiplatelet agents may have favored the “thrombization” of the pulsating hematoma in the periaortic site, not allowing the detection of flow signals in the color Doppler evaluation and preventing the typical arterial phase enhancement of the pseudoaneurysm by CT. On the other hand, some cardiosurgical materials may have reduced the diagnostic power of the CT scan, making, in this case, simple postoperative follow-up through TEE more accurate. In particular, during surgery with the Bentall procedure, in order to facilitate the closure of the surgical suture, much biological glue is used. Here, overlapping with the possible periaortic hematoma, the typical blood hyperdensity at baseline CT evaluation can be masked, providing periprosthetic structures with a hypodense aspect that can mislead the cardioradiologist.

## Conclusion

Interdisciplinary communication between cardiovascular radiologists and the cardiac surgery team is essential to improve diagnostic skills in identifying postoperative complications.

## Data Availability

Data sharing is not applicable to this article, because no datasets were generated or analyzed during the current study. Please contact the authors for data requests.
